# Rapid chromosomal evolution in the bush-cricket *Gonatoxiahelleri* Hemp, 2016 (Orthoptera, Phaneropterinae)

**DOI:** 10.3897/CompCytogen.v14i3.54422

**Published:** 2020-08-28

**Authors:** Elżbieta Warchałowska-Śliwa, Beata Grzywacz, Anna Maryańska-Nadachowska, Klaus-Gerhard Heller, Claudia Hemp

**Affiliations:** 1 Institute of Systematics and Evolution of Animals, Polish Academy of Sciences, Sławkowska 17, 31-016 Kraków, Poland Polish Academy of Sciences Kraków Poland; 2 Magdeburg, Germany Unaffiliated Magdeburg Germany; 3 University of Bayreuth, Dept. Plant Systematics, Bayreuth, Germany University of Bayreuth Bayreuth Germany

**Keywords:** 18S rDNA, adaptive radiation, C-banding, chromosome rearrangements, FISH, fluorochrome staining, NOR, Phaneropterinae, Tanzania, telomeric DNA

## Abstract

*Gonatoxiahelleri* Hemp, 2016 is one of the most widespread bush-crickets of the genus *Gonatoxia* Karsch, 1889 in East Africa. This species with seven large chromosomes (2n♂ = 7) differs from other representatives of the genus *Gonatoxia* drastically by its reduced chromosome number, the asymmetrical karyotype including karyomorphs rarely found in tettigoniids, as well as in irregularities in the course of meiosis. To better understand the origin of such an exceptional karyotype, chromosomes of 29 specimens from four populations/localities were studied using classical techniques, such as C-banding, silver impregnation, fluorochrome double staining and fluorescence *in situ* hybridization (FISH) technique with 18S rDNA and (TTAGG)*_n_* telomeric probes. FISH showed many 18S rDNA loci as well as interstitial telomeric sequences, where chromosome morphology varied in these components in terms of quantity and distribution. The 18S rDNA loci coincided with active NORs and C-banding patterns. We suggest that a combination of Robertsonian rearrangements and/or multiple common tandem fusions involving the same chromosomes contributed to the formation of this karyotype/karyomorphs. The results are the first step towards a better understanding of chromosomal reorganization and evolution within the genus *Gonatoxia*. Low chromosome number, together with the incidence of chromosomal polymorphism that is higher in *G.helleri* than previously reported in bush-crickets, implies that this species can be a valuable new model for cytogenetic and speciation studies. Our findings suggest that chromosomal translocations lead to diversification and speciation in this species and could be the driving force of adaptive radiation.

## Introduction

Chromosome number and structure, including their size and morphology, are important aspects of genome organization, because chromosomal variation may lead to species divergence. The analysis of the karyotype is also a useful feature in the systematic and evolutionary analysis because closely related species tend to have more similar karyotypes than more distinctly related ones (Sumner 2003). Changes in chromosome numbers (karyotype variability) or chromosome polymorphism within species as observed in many plant and animal groups may be involved in adaptation (e.g. Potter et al. 2017). The role of chromosomal rearrangements (translocations, inversions, changes in chromosome number) in the formation of reproductive barriers has been investigated and found to play a causal role in the isolation of species or populations in some genera of Hemiptera (Mills and Cook 2014, Chirino et al. 2017), Diptera (Coluzzi et al. 2002), Coleoptera (Kobayashi et al. 2000, Xavier et al. 2018), Lepidoptera (Vershinina and Lukhtanov 2017, Lucek 2018), and Orthoptera (e.g. Kawakami et al. 2011, Taffarel et al. 2015, Buleu et al. 2019, Silva et al. 2019).

Comparative cytogenetics, as a powerful tool to study karyotype variation, is based on accurate chromosome identification. Physical mapping involves fluorescence *in situ* hybridization (FISH) of specific segments of genomic DNA to their physical location on chromosomes, and it is useful in terms of gaining an insight into structural arrangements within the genome. The presence of repetitive DNA clusters in some genomic regions may represent fragile breakage sites that are associated with rearrangements during chromosome evolution (e.g. Schneider et al. 2013). Recently, series of works with FISH and conventional chromosome banding showed that the number and location of rDNA and heterochromatin sites can be useful markers for the study of tettigoniid karyotype evolution, and for the identification of genus/species-specific patterns (e.g. Grzywacz et al. 2011; Warchałowska-Śliwa et al. 2011, 2013a; Grzywacz et al. 2014a, b).

East Africa is a region of exceptional diversity of Orthoptera including Tettigoniidae bush-crickets (e.g. Hemp et al. 2013a, b, 2017). In the last few years, numerous papers have been published about East African Phaneropterinae taxa including descriptions of new genera and species combined with genetic studies, mainly on chromosome level (e.g. Hemp et al. 2010a, b, 2014, 2015a, b, c; Warchałowska-Śliwa et al. 2015; Hemp et al. 2016a, b, 2018). *Gonatoxia* Karsch, 1889 is a poorly known genus occurring in East Africa from which to date four species have been described. From *G.maculata* Karsch, 1889, little is yet known, although it is probably widely distributed throughout Tanzania, Kenya and Somalia, inhabiting deciduous dry forests and savanna woodlands in northern Tanzania; also little is known for *G.immaculata* Karsch, 1889, a species adapted to wet lowland forest in the East Usambara Mountains and along the Tanzanian coast; *G.furcata* Hemp, 2016 is probably endemic to the Udzungwa Mountains; *G.helleri* Hemp, 2016 was found syntopically at some localities with *G.immaculata* and *G.furcata*. The ecological niche of *G.helleri* seems to be broader than in the other species of the genus, as it occurs from coastal and lowland wet forests (e.g. East Usambara Mountains) up to montane elevations (e.g. Uluguru Mountains).

*Gonatoxia* is a very unusual genus within the subfamily Phaneropterinae, characterized by rarely observed high variability of chromosomes (both chromosome number and structure, 2n♂ = 7, 27 or 29) in bush-crickets (Hemp et al. 2016a). Our preliminary analysis of *Gonatoxia* showed that compared to other investigated East African Phaneropterinae, *Gonatoxiahelleri* had the lowest number of chromosomes (2n♂ = 7). Our cytogenetic studies indicated that such dramatic chromosomal rearrangements probably took place during a relatively early stage of speciation in *G.helleri* which is one of the most wide-spread and intriguing species of the genus so far (Hemp et al. 2016a). In the present study, a detailed cytogenetic characterization of *G.helleri* was performed using different techniques including mapping of repetitive DNA sequences characterizing chromosomal diversity. Based on the markers obtained we try to clarify the rearrangements responsible for intra- and inter-specimen chromosomal variability. It is important to investigate the potential role of repetitive DNA in the chromosomal evolution of this species.

## Material and methods

Cytogenetic analysis was conducted on 19 males and 10 females of *G.helleri* collected from four populations/localities in northern Tanzania: Morogoro District, Udzungwa Mountains [Ud], National Park Headquarters, Mangula Gate, lowland wet forest, 300 m (males: CH7949, CH8048, CH8087, CH8088, CH8089, CH8144, CH8145 CH8247; females: CH8072, CH8073, CH8138, CH8139, CH8146, CH8147), and Uluguru Mountains [Ul], forest above Morningside, 1800–2100 m (males: HE89, HE96, HE105, CH8246, CH8251, CH8252, CH8253; females: CH8250, CH8289) as well as East Usambara, Nilo [Ni] forest reserve, lowland wet to a submontane forest, 450–1150 m; (male CH8134, HE97, HE104; female CH8135) and Sigi Trail [Si], 450 m, East Usambara Mountains (male CH862; female CH8136)

Testes, ovaries, and somatic hepatic caeca were dissected, incubated in hypotonic solution (0.9% sodium citrate) and fixed in Carnoy’s solution [ethanol – acetic acid (3:1, *v/v*)], squashed in 45% acetic acid, followed by removal of coverslips using the dry ice technique and air-drying. For karyotyping and the identification of chromosome rearrangements, the preparations from all specimens were used for C-banding according to Sumner (1972). Additionally, some slides were analysed qualitatively by CMA_3_ (chromomycin A_3_) and DAPI (4,6-diamidino-2-phenylindole) staining (Schweizer 1976) as well as by AgNO_3_ (silver nitrate) staining to visualize active nucleolus organizer regions (NORs) (Warchałowska-Śliwa and Maryańska-Nadachowska 1992).

The best preparations (for individuals Ud: CH7949, CH8048, CH8088; Ul: HE89, HE96, CH8252; Si: CH621; Ni: HE97) were used for fluorescence *in situ* hybridization (FISH). All FISH experiments with 18S rDNA and telomeric probes were carried out according to the protocol described in Grzywacz et al. (2018). Unlabelled 18S rDNA probe was generated by PCR, using the genomic DNA of bush-crickets as templates. The probe was labelled with biotin-16-dUTP (Roche Diagnostics GmbH, Mannheim, Germany). The telomeric probe was generated by non-template PCR. The unlabelled telomeric probe was labelled with digoxigenin-11-dUTP (Roche Diagnostics GmbH). The detection of biotin-16-dUTP and digoxigenin-11-dUTP was carried out by avidin-FITC (Invitrogen, USA) and anti-digoxigenin rhodamine (Roche Diagnostics GmbH), respectively. Finally, the slides were counterstained with DAPI and mounted in the DABCO-based antifade solution. Preparations from FISH experiments were observed under a fluorescence microscope. Color images were recorded with a CCD DS-U1 camera using the NIS-Elements BR 3.0 software package. For each individual, at least 10 mitotic metaphase (oogonial/spermatogonial) and/or 20 meiotic divisions were analyzed using different markers.

## Results

**Figure 1.**
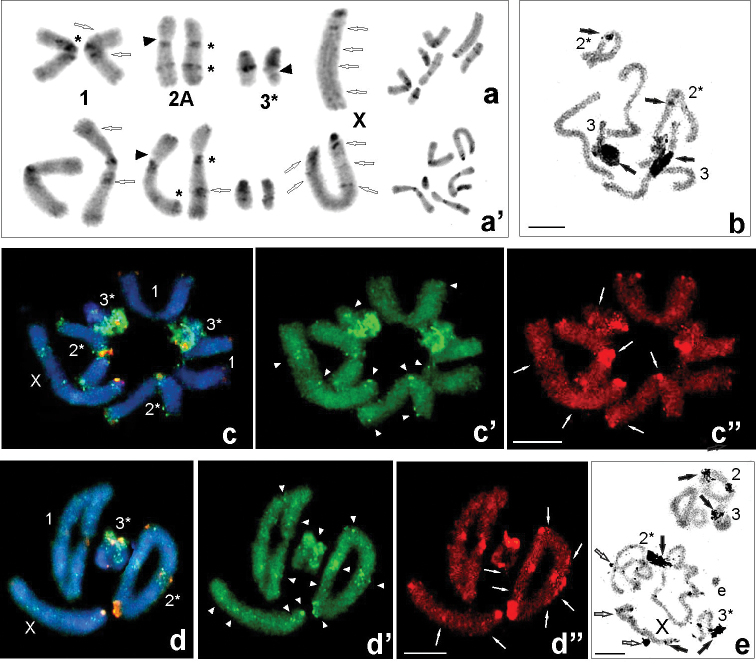
Examples of C-banding (**a, a**’), silver nitrate staining (**b, e**) and fluorescence *in situ* hybridization (**c–c**”, **d–d**”) in individuals with karyomorph 2A from populations: Udzungwa Mts (Ud CH8089) (**a)**, Sigi (Si CH8621) (**a**’), Nilo (Ni HE97) (**b, c–c”, d–d”, e)**. C-banding karyotypes of males chromosome complement (arranged from mitotic metaphase – right side); open arrows point to interstitial C-bands in the X chromosome and chromosome pairs 1^st^ and 2^nd^; black arrowheads indicate secondary constriction (**a, a**’). AgNO_3_ staining in male spermatogonial metaphases (**a**) and metaphase I/ diplotene (**e**) revealed medium sized and large active NORs of the bivalents 2^nd^ and 3^rd^ (black arrows) and very small NORs seen in the X (open arrows). FISH using 18S rDNA (green – **c, c’, d, d**’) and telomeric DNA (red – **c, c”, d, d**”) probes in mitotic metaphase (**c**) and metaphase I (**f)**; white arrowheads point to rDNA clusters near centromeric, interstitial and telomeric regions of the chromosomes (**c’, d**’) and white arrows ITS signals (**c”, d**”). Heterochromatin (**a, a’, b, e**) and hybridization areas (**c, d**) vary in size between homologous chromosomes, which are marked with asterisks (*). Elements (e) arisen from rearrangements were found (**e**). The X chromosome is indicated. Scale bars: 10 µm.

**Figure 2.**
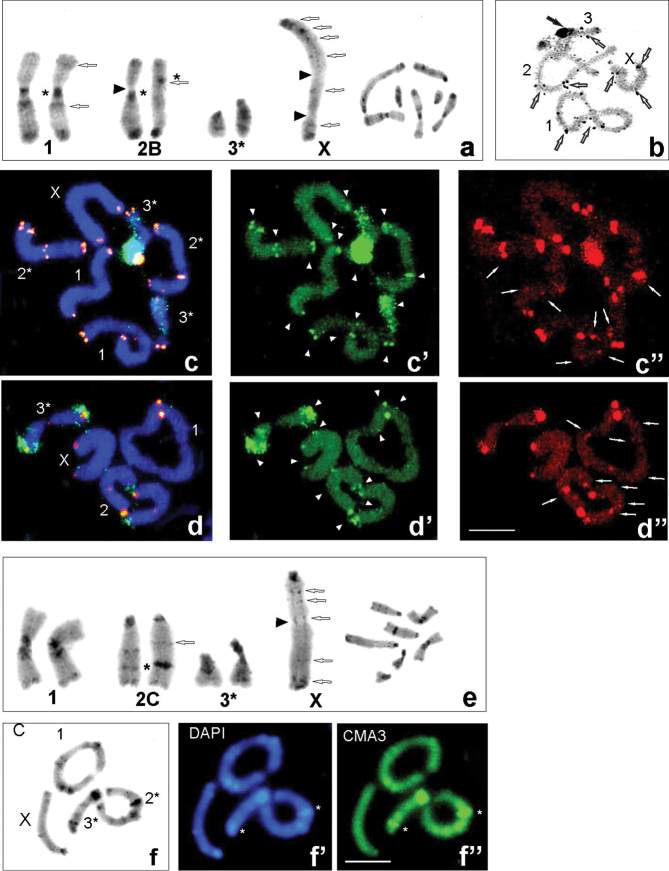
Examples of C-banding (**a, e**), silver nitrate staining (**b)**, C-, DAPI and CMA_3_ stained heterochromatin (**f–f**”) and FISH (**c–c”, d–d**”) in individuals with karyomorph 2B (**a–d**”) and 2C (**e, f**”) from populations: Udzungwa Mts (Ud CH7949 and CH8088) (**a, b)**, Ud CH8088 **(c–c**”), Uluguru Mts (Ul HE89) (**d–d”)**. C-banding karyotypes of males chromosome complement (arranged from mitotic metaphase – right side); open arrows point to interstitial C-bands in the X chromosome and chromosome pairs 1^st^ and 2^nd^; black arrowheads indicate secondary constriction (**a, e**). AgNO_3_ staining (**b**) at diplotene revealed large active NOR of the 3^rd^ bivalent (black arrows) and very small NORs seen in the X and bivalents (open arrows). FISH using 18S rDNA (green – **c, c’, d, d**’) and telomeric DNA (red – **c, c”, d, d**”) probes in mitotic metaphase (**c**) and diakinesis (**d)**; white arrowheads point to rDNA clusters near centromeric, interstitial and telomeric regions of the chromosomes (**c’, d**’) and white arrows ITS signals (**c”, d**”). C/DAPI/CMA_3_ blocks were located very close to each other, but bright CMA_3_ signals coincided with active NORs. Heterochromatin (**a, e, f**) and hybridization areas (**c, d**) vary in size between homologous chromosomes, which are marked with asterisks (*). The X chromosome is indicated. Scale bars: 10 µm.

**Figure 3.**
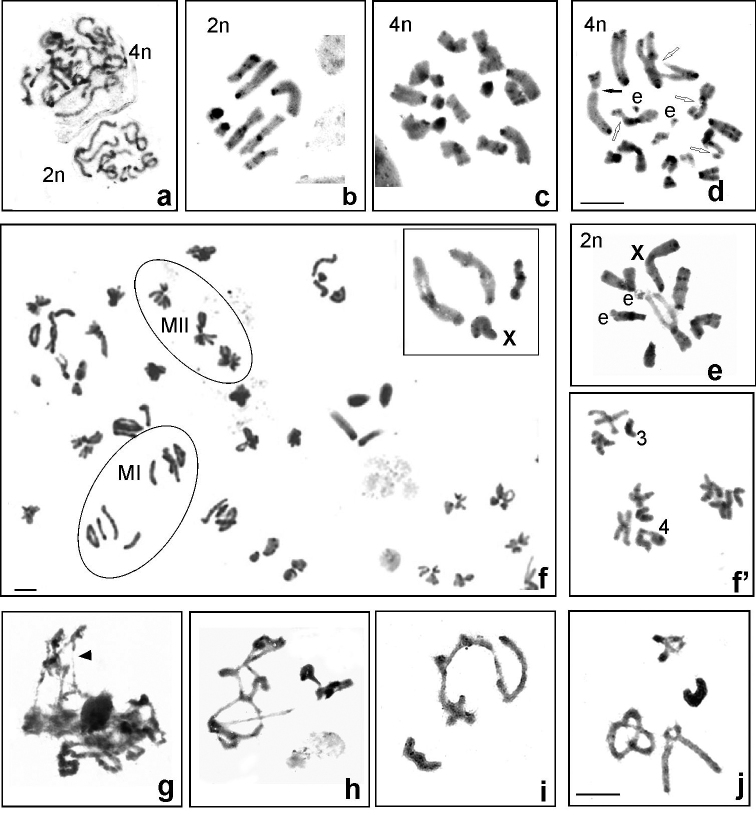
C-banded mitosis and meiosis (**a–j**). Spermatogonial early metaphase (Uluguru Mts [Ul] HE96) (**a)**, oogonial metaphase (Udzungwa Mts [Ud] CH8138) (**b, c, d)** and spermatogonial metaphase (Ud CH8088) (**e)** with diploid and tetraploid cells. Black arrow indicates secondary constriction and open arrows point of deletions in one of homologous chromosomes (**d).** Elements (e) resulting from rearrangements were found in both female and male cells (**d, e**). During meiosis, bivalents show crossing over in metaphase I (**f** – in the right corner) and normal metaphase II complements with 3 (3+0) or 4 (3+X) chromosomes (**f, f’)**. Arrowhead indicates asynapsis in early prophase I (**g).** In diplotene/diakinesis, a multivalent-like chain (**h**) or end-to-end association comprising three autosomal elements (**i)** as well as asynapsis in individuals Ul CH8246 (**g)** and Ud CH8088 (**h–j)** were observed. The X chromosome is indicated. Scale bars: 10 µm.

The study of mitotic metaphase spermatogonial, oogonial, and somatic gastric caeca cells showed 2n = 7 (6+X), FN = 10–13 in most cells of the male and 2n = 8 (6+XX), FN = 11–14 in the female. In the karyotype, the first long pair of autosomes was metacentric, whereas the second long (three main karyomorphs) and small third pairs (four main karyomorphs) were polymorphic with respect to the morphology of homologous chromosomes in specimens of the analyzed localities. The 2^nd^ chromosome pair showed three main karyomorphs: homozygous metacentric (2A) [18 specimens: Udzungwa (Ud) 9, Uluguru (Ul) 3, Nilo (Ni) 4, Sigi (Si) 2], heterozygous – subacro/ acro (2B) [9 specimens: Ud 5, Ul 4] and homozygous acrocentric (2C) [2 individuals from Ul]. The 3^rd^ chromosome pair was greatly polymorphic and was observed in both Ud and Ul populations. It should be noted that in individuals from Si and Ni populations (few individuals analyzed), the 1^st^ and 2^nd^ chromosome pairs were homozygous (both bi-armed) in terms of chromosome morphology. The acrocentric sex chromosome (X) was the largest element of the set (Figs 1–3).

**Figure 4.**
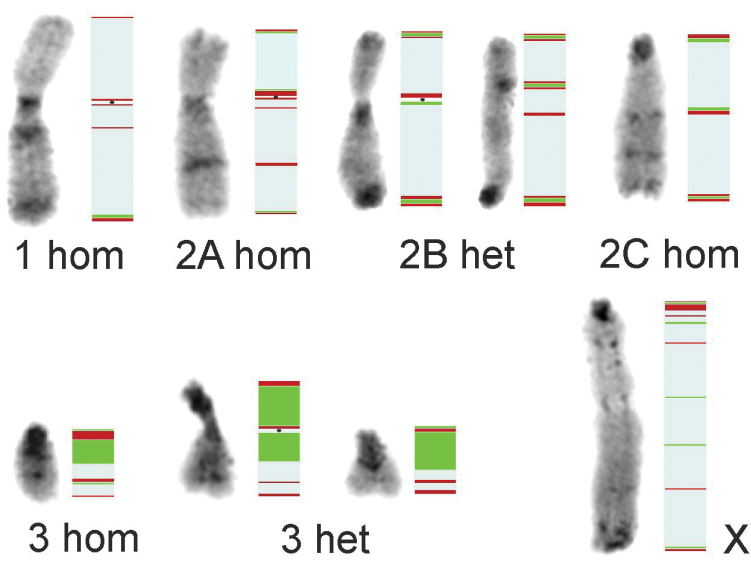
Scheme summary the distribution of C-banding pattern (represented in left side), 18S rDNA (green) and as well as true telomeres (at the ends) and interstitial telomeric (ITS – in the inner parts) repeats (red) of *Gonatoxiahelleri*. Three chromosome pairs (1^st^ and polymorphic 2^nd^ and 3^rd^) in main karyomorphs [homozygous metacentric (1A hom, 2A hom, 3 hom), heterozygous – submeta/ acrocentric (2B het, 3 het), homozygous acrocentric (2C hom)] and X chromosome showed differences in the size and position of rDNA and tDNA signals detected by FISH and generally demonstrate a coincidence between the location of rDNA loci and C-positive heterochromatin regions. The presence of ITSs near the pericentromeric and interstitial and/ or near telomeric region suggest that karyomorphs could be the result of different chromosomal rearrangements.

Constitutive heterochromatin blocks with pericentromeric thick C-bands were found in all chromosomes. Additionally, the bi-armed first pair possessed thin telomeric and two interstitial (near the centromeric region in one arm and thin near the end in the second arm) C-bands, which are a feature in distinguishing this pair from the 2^nd^ pair, more or less similar in size. The heterochromatin in the 1^st^ pair revealed a discrete size polymorphism in the C-patterns. Also, the 2^nd^ (karyomorphs 2A, 2B, 2C) and 3^rd^ chromosome pairs showed heteromorphism in terms of the size/locality of bands on respective homologous chromosomes. Pericentromeric, interstitial and terminal C-bands with differences in size were observed on the acrocentric X chromosome (Figs 1a, a’, 2a, e, 4). The secondary constriction (not always seen) of the 2^nd^ chromosome and the X chromosome were located near the C-band (Figs 1a, a’, 2a, e). Besides a large active NOR in 3^rd^ chromosomes/bivalent and a smaller in the 2^nd^, being coincident with secondary constrictions, a site with faint silver nitrate staining was observed, indicating the occurrence of small NORs, probably “secondary NORs”, in bivalents and the X chromosome (Figs 1b, e, 2b). Positive C-blocks in the 1^st^ chromosome pair were neutral for G+C (DAPI+) and A+T (CMA_3_+) base pairs, whereas the 2^nd^ and 3^rd^ pair of chromosomes with interstitial C-bands revealed clearly seen CMA_3_+ block (for example Fig. 2f, f’, f”) which coincided with active NORs.

Physical mapping by FISH with the 18S rDNA and telomeric probes was performed in eight individuals from four analyzed populations (Ud: CH7949, CH8048, CH8088; Ul: HE89, HE96, CH8252; Si: CH621; Ni: HE97). Generally, all examined specimens demonstrated similar rDNA signals located in the centromeric, interstitial and telomeric regions and usually were connected with C-positive regions. The acrocentric/ subacrocentric 3^rd^ chromosome pair carried major rDNA located near the centromeric region and interstitial minor 18S rDNA clusters. Additionally, low-intensity/small clusters on the 2^nd^ chromosome pair and the X chromosome, both near the centromeric regions were observed (Figs 2c, c’, d, d’, 3c, c’, d, d’, 4). Heteromorphism of rDNA-signals (marked with an asterisk), similar to C-heterochromatin bands (Figs 1a, a’, 2a), was observed in terms of the size/ strength or presence/ absence on the homologous chromosome depending on the karyomorphs in the 1^st^, 2^nd^ and 3^rd^ chromosome pairs. Some interstitial C-positive regions in the sex chromosome contained rDNA (Figs 1c’, d’, 2c’,d’, 4).

FISH analyses with the (TTAGG)*_n_* probe generated signals in telomeres of all chromosomes but the size of the clusters on different arms of some chromosomes and between individuals, with different karyomorphs varied as well (Figs 1c”, d”, 2c”, d”). In addition to the typical telomeric, of the so-called interstitial telomeric sequences (ITSs) in the inner parts of all chromosomes were observed at both mitotic metaphase (Figs 1c”, 2c”) and diakinesis/ metaphase I (Figs 1d”, 2d”). The signals within secondary constrictions were much larger than those at the chromosome ends. The 2^nd^ chromosome pair in the karyomorph A exhibited heteromorphic signals of ITS in both the bi-armed chromosomes in the centromeric region (Fig. 1c”, d”). Whereas bright hybridization signals were detected in the centromeric region in the bi-armed homologue and interstitially located in the acrocentric homologue of the 2^nd^ pair in karyomorph B (Fig. 2c”, d”). Besides that, additional week ITS signals, present in a low copy number, in subterminal/ medial position in autosomes and sex chromosome were observed (Figs 1c”, d”, 4).

In some individuals with a chromosome number close to the diploid count, intra-individual variability cells with 14 (male) and 16 (female) chromosomes were observed, probably corresponding to tetraploid levels. Thus, based on the analysis of 50 metaphase cells per individual, about 11% oogonial (Ud: CH8073, CH8138, CH8147, Ni: CH8135, Ul: CH8250) and about 5% spermatogonial cells (Ud: CH7949, CH8048, CH8088, CH8145, Ul: HE89, HE96, HE105, CH8251, CH8251, Ni: CH8134) had tetraploid chromosome numbers (Fig. 3a, c, d). However, in somatic gastric caeca mitotic metaphase in both male (Ud: CH7949, CH8088, Ul: CH8252, Si: CH8621) and female (Ud: CH8146, CH8147, Ul: CH8289, Ni: CH8135) cells with intra-individual variability of chromosome numbers were not observed.

Cytogenetic preparations of the females did not show cells in meiotic division. Male meiosis was classified as synaptic and chiasmate because the chromosomes were generally paired during early pachytene stage and bivalents exhibited a chromosomal configuration indicating crossing over (Fig. 3f upper right corner). In anaphase I, the X chromosome segregated to one pole and in metaphase II, there were 3 (3+0) or 4 (3+X) chromosomes, confirming the regular segregation of all chromosomes during the first meiotic division. (Fig. 3f, f’). However, in some specimens from different populations: Ud (CH8088, CH8089, CH8144, CH8138, CH8147, CH8247), Ni (HE97) and Ul (HE96, CH8246) sporadically in pachytene spermatocytes, the chromosomal elements showed asynapsed interstitial and/ or terminal chromosomal segments (Fig. 3g, j) and in the prophase I a multivalent-like chain or end-to-end association comprising three autosomal elements (Fig. 3h, i). Furthermore, lightly stained, less condensed chromatin in some regions on the bivalents (chromosome pair 3^rd^) or one of homologous chromosomes in mitotic metaphase, deletions, or fragments of chromosomes/ elements were observed in both male (Fig. 3d, j) and female (Fig. 3e) cells. Additionally, in these populations, very rarely mitotic spermatogonial cells showed intra-individual variability since one autosome was lacking.

## Discussion

The result obtained here is in accordance with the previous study about diploid chromosome numbers in *Gonatoxiahelleri* (Hemp et al. 2016a). It is probably a young species with specific karyotypic macrostructure, a very interesting case with the lowest number of chromosomes (2n♂ = 7, X0 type of sex determination) compared to other species of the genus *Gonatoxia* (2n♂ = 29, 27, X0) and Phaneropterinae as a whole. Such a low number of chromosomes, in addition to morphological differences, allowed describing the analyzed individuals as a new species in this genus (Hemp et al. 2016a). To date, a similar karyotype has been described only in the Australian *Yutjuwaliasallyae* Rentz, 2001 (Listroscelidinae), with 7 metacentric chromosomes in the male, but without additional information (Ueshima 2001). Most East African bush-crickets of Phaneropterinae, e.g. *Parapyrrhicia* Brunner von Wattenwyl, 1891, *Eurycorypha* Stål, 1873, and *Plangia* Stål, 1873 have uniform karyotypes (Hemp et al. 2013a, 2015a, 2017) with a diploid chromosome number (2n♂) 31 or 29 and the X0 type of sex determination.

The main characteristic of the karyotype of *G.helleri* is the presence of very large autosomes compared to the other species of this genus, based on the analysis of the main relative lengths of the autosomes (Warchałowska-Śliwa et al. in preparation). This reflects the derivation from multiple rearrangements. Even under the assumption that fusions or inversions are frequent in orthopteran chromosomal evolutionary history (e.g. White 1973, Hewitt 1979, Warchałowska-Śliwa 1998) leading to a reduced diploid number, the karyotype of *G.helleri* is an extreme example compared to other phaneropterines and *Gonatoxia* species (Warchałowska-Śliwa et al. in preparation). In specimens analyzed in this paper, probably Robertsonian translocations (centric fusion) and tandem fusions and/or inversion were the reason of the observed morphological arrangements in the 1^st^ chromosome pair in all investigated populations of *G.helleri*. However, in the 2^nd^ chromosome pair, such types of chromosomal rearrangements occurred only in the Sigi (Si) and Nilo (Ni) populations of the species. In some individuals, the 2^nd^ chromosome pair [Udzungwa Mts (Ud) and Uluguru Mts (Ul)] and the 3^rd^ chromosome pair (Ud, Ul, and Ni) were polymorphic with respect to the morphology (bi-armed or acrocentric) of homologous chromosomes. Unfortunately, not much can be said about the variability of the karyotype in individuals from the Sigi population due to the insufficient number of individuals analyzed. Robertsonian translocations are the basic mechanism of rearrangements of chromosomes in the evolution of the orthopteran karyotype, especially in Acrididae grasshoppers (e.g. Taffarel et al. 2015) and some katydids Tettigoniinae, Saginae and Bradyporinae (Warchałowska-Śliwa et al. 2009, 2013a; Grzywacz et al. 2017). These rearrangements are not usually found in Phaneropterinae, as here tandem fusions dominate in karyotype evolution (e.g. Warchałowska-Śliwa 1998). Due to such extensive reorganization, it is very difficult to determine the order and evolutionary causes of these specific rearrangements that led to the extreme karyotype evolution of *G.helleri*, compared to the probable ancestral number of chromosomes (2n♂ = 29) in the genus *Gonatoxia*.

Physical mapping involving fluorescence *in situ* hybridization of specific segments of genomic DNA is extremely useful in terms of making an insight into structural rearrangements within the genome. Repetitive sequences change rapidly during evolution, providing excellent markers for the identification of chromosomes, chromosome segments and the resulting evolutionary chromosome rearrangements (e.g. in crickets: Palacios-Gimenez et al. 2015). Generally, Phaneropterinae usually carry one rDNA/ NOR (per haploid genome), found in the pericentromeric region or rarely located interstitially (e.g. Grzywacz et al. 2011; Warchałowska-Śliwa et al. 2011, 2013b; Grzywacz et. al. 2014a, b). In other orthopterans, a high number and variation of rDNA was observed mainly in the grasshopper taxa *Podismasapporensis* Shiraki, 1910 and *P.pedestris* (Linnaeus, 1758) (Veltsos et al. 2009, Grzywacz et al. 2019), *Eyprepocnemisplorans* (Charpentier, 1825) (Cabrero et al. 2003), or *Abracrisflavolineata* (De Geer, 1773) (Ferretti et al. 2019). Compared to phaneropterines and other tettigoniids, the chromosomal distribution of major 18S rDNA in *G.helleri* described in this paper is unique because: (i) it occurs in a high number of sites with different intensity of hybridization signals, (ii) it is located pericentromerically, interstitially and near the telomeric regions, (iii) clusters of various size or heteromorphic structures occur in the same chromosome pair. The 3^rd^ chromosome pair (most variable in the amount of heterochromatin) carried large/ high intensity clusters near the pericentromeric region, whereas in the 2^nd^ chromosome pair and the X chromosome this cluster was very small. Additionally, interstitial regions with a very low intensity of hybridization signals of 18S rDNA were seen located in different chromosomes (Fig. 4). Generally, our results demonstrate a coincidence between the location of rDNA loci, C-positive (Fig. 4) and GC-rich heterochromatin regions as well as active NORs (occurring in secondary constrictions and small ones in bivalents and the X chromosome). It should also be noted that heterochromatin amplification or loss could be responsible for the variation in the morphology among karyomorphs in all three pairs of chromosomes. In some grasshoppers a large variety of localization of 18 rDNA and tDNA clusters is not associated with structural rearrangements of chromosomes in the karyotype but to the evolution/massive amplification of repetitive DNA (Bugrov et al. 2016, Buleu et al. 2019).

Both conventional heterochromatin staining and rDNA-FISH revealed size heteromorphism/polymorphism between homologous chromosomes, indicating either recent or rapid evolution in this species. The presence of individuals with heteromorphic pairs may be a result by unequal meiotic cross-over, tandem duplication of ribosomal sequences and related to sister chromatid exchange or translocation rearrangements or homologous recombination (e.g. Cabral-de-Mello et al. 2011; Warchałowska-Śliwa et al. 2013a,b; Grzywacz et al. 2014a, b).

Another universal probe is the telomeric sequence [tDNA, (TTAGG)*_n_*]) that itself is an ideal marker for the identification of chromosome ends (Kuznetsova et al. 2020) and a marker for chromosome rearrangements, being conserved in many groups of insects (Vítková et al. 2005). Interstitial telomeric sequences (ITSs) reflect remnants of multiple chromosome fusions of ancestral chromosomes e.g. in moths (Lepidoptera, Noctuidae: Rego and Marec 2003), giant water bugs (Hemiptera: Chirino et al. 2017) or grasshoppers (Jetybayev et al. 2012, Grzywacz et al. 2019). The individuals examined in this study showed differences in the intensity and position of the hybridization signals of the (TTAGG)*_n_* probes in both autosomes and sex chromosomes (Fig. 4). Variation in the intensity of signals in chromosomes, including sex chromosomes, could result from differences in the length of target TTAGG sequences. Sometimes the lack of hybridization signals in some ends of chromosomes suggests a low number of copies of telomeric repeats (López-Fernández et al. 2004).

The karyotype described here for *G.helleri* is different from that described for other species of this genus since we found a reduction in the number of acrocentric pairs (Hemp et al. 2016a), and probably an inter-population variation (more individuals should be analyzed). In this species several sites with ITS were identified in addition to the terminal/true telomeric sequences. The presence of ITSs near the pericentromeric and interstitial and/ or near telomeric region (Fig. 4) suggest that this karyotype /these karyomorphs could be the result of telomere-telomere fusions of the chromosomes, inversions (intra-chromosomal rearrangements), unequal crossing over, or the insertion of telomeric DNA into unstable sites during the repair of double-strand breaks (e.g. Bolzán and Bianchi 2006). ITS repeats in the pericentric C-positive block of the bi-armed chromosome pairs in the 2^nd^ and the smaller 1^st^ chromosome pairs of *G.helleri* created through centric fusion has never been recorded in any phaneropterine so far. Thus, it cannot be excluded that these centric fusions are not the result of Robertsonian rearrangements (i.e. fusion-fission cycle) but are true fusions that left remnants of telomeric DNA in the arms of the acrocentric chromosomes involved in the fusion. A similar observation was described in the grasshopper species *Chorthippusjacobsoni* (Jetybayev et al. 2012). In this case, the chromosome break points are localized near the centromere, which are less frequently involved in the formation of dicentric chromosomes. Additionally, it is also worth noting that the acrocentric X chromosome with some interstitial ITSs thin C-bands (Figs 1c’, d’, 4) and sometimes secondary NORs (Figs 1a, a’, e, 2a, b, e) might have undergone sequential inversions and/or end-to-end fusions during the chromosome evolution of *G.helleri*.

Some individuals of *G.helleri* exhibited multivalent chromosome associations during meiosis I, asynapsed and/or heterosynapsed chromosome segments and bivalents with distinctly associated regions (gaps and less condensed chromatin) in postpachytene nuclei. These findings indicate that certain chromosome regions were non-homologous and carried heterozygous chromosomal rearrangements. Various degrees of heterosynapsis/asynapsis have also been described in other organisms, which were heterozygous for paracentric or pericentric inversions in grasshoppers or scorpions (e.g. Díez and Santos 1993, Mattos et al. 2013). Variation as the chromosome polymorphism observed in *G.helleri* indicates that the meiotic segregation of these chromosomes has not led to the production of gametes with unbalanced chromosomes and consequent fertility loss. It suggests that some of the chromosome mutations had no negative impact on the carriers and were neutral or may have even increased fitness (Menezes et al. 2013). In *G.helleri*, based on the orientation of the chromosomes in metaphase I and the haploid complement observed in metaphase II, the chromosomes analyzed in the present work probably underwent balanced segregation. A fascinating intra-individual variability of chromosome numbers in some mitotic spermatocytes/oogonia and in meiotic cells was observed as tetraploid cells. This observation could indicate the occurrence of endopolyploidy. The absence of polyploid cells in metaphase II nuclei can be assumed that the “aberrant cells” degenerated during the cellular cycle as errors in chromosome segregation (Mattos et al. 2013). The formation of polyploid mitotic cells can be probably associated with problems in cytokinesis that disrupt chromosome segregation.

Based on the results presented in this paper, we suggest that the change in chromosome numbers associated with multiple chromosomal rearrangements and observed heterozygous chromosomes may have presented a precondition to colonize new habitats and might be a case of adaptive radiation in *G.helleri*. Generally, the occurrence of chromosomal changes may be the result of ancestral allopatry, sympatry, and/or hybridization (meiotic and mitotic instability), demographic processes associated with colonization (founder effect), environmental fragmentation or a combination of these factors, and it may also point to recent speciation processes and hybridization (e.g. Dion-Côté et al. 2017; Gould et al. 2017). An excellent example for the role of chromosomal rearrangement in speciation in orthopteran insects is the Australian genus of morabinae grasshoppers *Vandiemenella* Key, 1976, specifically the *viatica*-species group. All taxa of this genus show extensive chromosomal variation, parapatric distribution patterns, and narrow zones at their boundaries. A number of population genetic and phylogenetic studies showed “extensive non-monophyly of chromosome races and suggest that geographical isolation leading to the fixation of chromosomal variants in different geographic regions, followed by secondary contact, resulted in the present day parapatric distribution of chromosomal races” (review Kawakami et al. 2011).

*Gonatoxiahelleri* is the only *Gonatoxia* species able to inhabit almost the complete offer of ecological niche forests in eastern Africa, while most other species of this genus are restricted to certain forested types. Many or maybe even most bush-cricket taxa probably were first forest dwellers and later adapted to open land habitats in Africa (Voje et al. 2009) due to the beginning aridification of Africa about 8 Mya (deMenocal 2004; Cohen et al. 2007). It cannot be excluded that inter-population differences in the genome may be a sign that species in high elevations of the Uluguru Mts and Udzungwa Mts have a different karyomorph compared to lowland populations of *G.helleri* along the coast, East and West Usambara (Nilo and Sigi populations). On the other hand, there may already have been a selection of different populations because they are now isolated from each other. Our results suggest that forest dwelling *Gonatoxia* species with a restricted area of occurrence, such as *G.furcata* and *G.immaculata*, are more basal taxa, and species which are wide-spread and inhabiting a broad ecological niche, such as *G.maculata* and *G.helleri*, might be evolutionary more recent species. *Gonatoxiahelleri* is probably the most plastic and adaptive species as well as has the most dramatically rearranged genome and inter-specific differences even within one population and might be the youngest species of the genus.

In conclusion, the cytogenetic analysis of *G.helleri* provides a new example of chromosomal evolution by multiple rearrangements. Several rearrangements, probably including primary (insertion, deletion or duplication, peri- or paracentric inversion, and intra- or interchromosomal reciprocal translocation) or secondary translocations were responsible for the formation of the karyotype and karyomorphs in *G.helleri*. The bi-armed chromosomes of the 1^st^ pair occurring in individuals from all populations probably originated by Robertsonian fusion, whereas the other two pairs in the set are still subject to continuous rearrangements. At this moment, the insufficient number of individuals analyzed from the Nilo Forest Reserve and the Sigi Trail populations in the East Usambaras does not allow to determine possible differences of individuals within and between the populations. Nevertheless, we suggest that these chromosome mutations had no negative impact on the fitness of carriers.

The present study demonstrates that molecular cytogenetic techniques as useful tools for understanding chromosomal organization and evolutionary history in the genus *Gonatoxia*. The chromosome number is lower and the degree of chromosomal polymorphism is greater in *G.helleri* than previously reported in bush-crickets. Our results suggest that this species may be a valuable new model system for further studying the potential role of morphological rearrangements of chromosomes in speciation. We determine the possibility that chromosomal rearrangements might be a driver of adaptive radiation enabling a species to broaden its ecological niche and thus higher adaptability to changing climatic conditions. The adaptive significance of chromosomal rearrangements for *G.helleri* and the origin of such low diploid chromosome numbers require additional genetic analyses, especially the development of multiple cytogenetic markers and molecular studies.
